# Bayesian Approaches in Drug Development: Interpreting the 2026 US Food and Drug Administration Draft Guidance for Optimal Dose Finding

**DOI:** 10.1002/pst.70149

**Published:** 2026-11-13

**Authors:** Ayon Mukherjee

**Affiliations:** ^1^ Population Health Sciences Institute Newcastle University Newcastle Upon Tyne UK

**Keywords:** Bayesian borrowing, Bayesian methodology, FDA draft guidance, project optimus, Type‐I error calibration

## Abstract

The US Food and Drug Administration (FDA) released a landmark draft guidance in January 2026 entitled ‘Use of Bayesian Methodology in Clinical Trials of Drug and Biological Products’ (Food and Drug Administration 2026)—the first comprehensive regulatory framework for Bayesian primary inference in drug and biologic trials. This Viewpoint critically appraises the Guidance across four dimensions: (1) The regulatory and philosophical tensions in Section IV.A.1, ‘Calibration to Type‐I Error Rate’, with specific improvement recommendations; (2) the Bayesian borrowing framework articulated in Sections III and V, including gaps in dynamic borrowing and effective sample size (ESS) interpretation; (3) the Guidance's synergy with FDA's Project Optimus and the companion dose‐optimisation guidance (August 2024) (Food and Drug Administration 2024), and how Bayesian methods underpin the optimal biological dose (OBD) paradigm; and (4) the limited operational detail in Section III.F for efficacy–toxicity joint models and seamless Phase I/II designs—the design class most capable of OBD identification. It is contended that the Guidance is a historic advance, yet its oncology dose‐finding section devotes most of its operational detail to maximum tolerated dose (MTD)‐oriented designs. A regulatory taxonomy of Bayesian dose‐finding designs and targeted improvements for the final guidance are proposed. The appraisal refers to the nonbinding January 2026 draft text, which may change before finalisation.

**Abbreviations**
ADC: Antibody–drug conjugate
BHM: Bayesian hierarchical model
BLA: biologics licence application
BLRM: Bayesian logistic regression model
BOIN: Bayesian optimal interval (design)
BOIN12: Bayesian optimal interval Phase I/II design
BOIN‐ET: Bayesian optimal interval–efficacy and toxicity design
CBER: Center for Biologics Evaluation and Research
CDER: Center for Drug Evaluation and Research
CID: Complex Innovative Trial Design
CRM: continual reassessment method
DLT: dose‐limiting toxicity
EffTox: efficacy–toxicity (joint model design)
EMA: European Medicines Agency
ESS: effective sample size
FDA: US Food and Drug Administration
FWER: Familywise Type‐I error rate
GBM: glioblastoma multiforme
ICH: International Council for Harmonisation
IND: investigational new drug
JAGS: just another gibbs sampler
MAP: meta‐analytic‐predictive (prior)
MCMC: Markov chain Monte Carlo
MCSE: Monte Carlo standard error
MSE: mean squared error
MTD: maximum tolerated dose
mTPI: modified toxicity probability interval
mTPI2: modified toxicity probability interval 2
OBD: optimal biological dose
PK/PD: pharmacokinetics/pharmacodynamics
POCRM: partial‐order continual reassessment method
SAP: statistical analysis plan
SAS: statistical analysis system
STEIN: simple toxicity and efficacy interval (design)
TITE: time‐to‐event (extended design framework)
TITE‐BOIN12: time‐to‐event BOIN12 design

## 1 Introduction

Bayesian methods offer well‐recognised advantages in clinical drug development: formal incorporation of prior knowledge, adaptive inference as data accumulate, synthesis of heterogeneous evidence and uncertainty quantification aligned with clinical decision‐making [1, 2]. Regulatory acceptance of Bayesian primary analyses has been most explicit for medical devices, where FDA issued dedicated guidance in 2010 [3]. The FDA's January 2026 draft guidance [4] substantially reduces the remaining uncertainty for drugs and biologics, establishing the first comprehensive regulatory framework for Bayesian inference across prior specification, borrowing, success criteria, operating characteristics, estimands and documentation. Jointly developed by CDER and CBER, the Guidance signals that Bayesian primary analyses are acceptable, provided designs carry principled priors, explicit success criteria, prospective simulation and computational transparency [5]. The Guidance is a draft (Docket FDA‐2025‐D‐3217) containing nonbinding recommendations: its use of ‘should’ denotes a suggestion rather than a requirement. This appraisal therefore refers to the January 2026 text, whose wording, section numbering, and examples may change in the final guidance; where ‘require’ or ‘recommend’ is used below, the reference is to proposals for the final guidance rather than to requirements of the draft.

The Guidance arrives at a pivotal juncture. Since 2021, FDA's Project Optimus has sought to replace the MTD paradigm with an OBD that maximises benefit–risk [6, 7], intensifying demand for Bayesian adaptive designs jointly modelling efficacy and toxicity. The Guidance is critically appraised across four themes: Bayesian borrowing; Section IV.A.1 Type‐I error calibration; Project Optimus complementarity; and the regulatory gap most consequential for OBD‐finding designs.

## 2 Overview of the 2026 Draft Guidance

The Guidance adopts a pluralistic framework for Bayesian success criteria: (1) calibration to frequentist familywise Type‐I error rate (FWER; Section IV.A.1); (2) direct interpretation of posterior probability, appropriate when the prior accurately reflects pre‐trial belief or external data (Section IV.A.2); and (3) benefit–risk or decision‐theoretic criteria (Section IV.A.3) [4]. Its most operationally significant contributions are detailed prior‐distribution specification, comprehensive Bayesian borrowing guidance illustrated with regulatory examples, and detailed expectations for simulation‐based evaluation of operating characteristics. Table 1 summarises key sections and regulatory messages.

**TABLE 1:** Key sections and regulatory messages in the FDA's 2026 draft guidance on Bayesian methodology.

Section	Topic	Key regulatory message
II	Background and definitions	Prior, likelihood, posterior, credible interval established as the regulatory lexicon
III.A–E	Bayesian borrowing (previous trials, concurrent‐control augmentation, paediatric extrapolation, similar diseases/basket trials, subgroups)	Regulatory examples provided; discounting approaches (power, commensurate, supervised power, mixture and elastic priors; BHMs) discussed in V.D; relevance of external evidence to be justified and documented (VIII.A).
III.F	Dose‐finding in oncology	CRM, BLRM, BOIN, mTPI and mTPI2 described for MTD finding; the Thall–Cook (EffTox) and BOIN12 designs cited for optimised dosages, without operationalised evaluation criteria in the draft text.
IV.A.1	Calibration to Type‐I error rate	Posterior threshold c chosen to control overall FWER, typically 0.025 one‐sided; for complex designs c is approximated by clinical trial simulation
IV.A.2–3	Direct posterior/decision‐theoretic	Direct posterior interpretation appropriate when the prior accurately reflects pre‐trial belief or external data; benefit–risk and loss‐function criteria described
V	Prior distributions	Prior specification, discounting of external information and prior‐influence metrics (e.g., ESS) discussed; sensitivity analyses to be planned (V.F)
VII–VIII	Software, documentation	Reliable software and sampling (VII); documentation of plans, simulation code, MCMC settings and diagnostics, software version details and seeds for chain initiation (VIII)

*Note:* The table summarises the January 2026 draft text; the Guidance is nonbinding and phrases its recommendations with ‘should’.

## 3 Bayesian Borrowing: Strengths and Critical Gaps

Bayesian borrowing—formally incorporating external data (historical trials, nonconcurrent controls, related populations or prior development phases) into the current trial prior—is the mechanism by which Bayesian methods most markedly diverge from frequentist inference [8, 9, 10]. The Guidance organises borrowing into five categories: prior‐trial borrowing (III.A); augmentation of a concurrent control (III.B); paediatric extrapolation (III.C); borrowing across similar diseases or subtypes, including basket trials (III.D); and BHM‐based analysis of subgroups (III.E) [4], illustrated with regulatory examples (REBYOTA, GBM AGILE, Precision Promise and paediatric empagliflozin/linagliptin supplements). Section V.D.4 describes static discounting through power priors, dynamic discounting through commensurate and supervised power priors, mixture priors, BHMs and elastic priors [4]. The Guidance does not name the meta‐analytic‐predictive (MAP) prior [11] or its robust mixture extension [12], which are nevertheless widely used to summarise historical control information and to protect against prior–data conflict, and which are discussed in recent commentary on the Guidance [5]. Together with power priors [13] and BHMs [14], these formulations span the practical range from fixed discounting to fully data‐driven shrinkage.

Two points warrant attention. First, Section V.D.4 describes dynamic discounting in general terms, noting that such methods borrow less when the data are less similar and that they introduce additional parameters to be specified, and it names commensurate and supervised power priors without citing the primary literature on commensurate or power priors. The foundational methodology—the commensurate prior [15] and power priors in which the discounting parameter is itself learned from the data [9, 16]—automatically scales borrowing to observed prior–data consistency. This is a gap in citation and operational specificity rather than in conceptual coverage: a sponsor consulting the Guidance is not directed to the primary methodological literature or to simulation‐based criteria for choosing the adaptivity mechanism. Such mechanisms are most relevant when exchangeability between external and current data is uncertain, as in the paediatric‐extrapolation and similar‐disease settings addressed in Sections III.C and III.D of the Guidance; the final guidance could therefore cite these foundational references and recommend pre‐specification and simulation‐based evaluation of the adaptivity mechanism that governs how borrowed information is downweighted as prior–data conflict increases. Second, Section V.E recommends that the influence of the prior be discussed and documented using metrics such as the prior mean and the ESS [17, 18], and advises against Type‐I error inflation as a measure of prior influence. These metrics describe the prior itself—its location and strength—but not how a mislocated high‐ESS prior affects the bias and mean squared error (MSE) of the final estimate, which can be severe. Because Section VIII.A already lists bias and MSE among the operating characteristics to be documented, the final guidance could make explicit that they should be examined under prior–data conflict alongside ESS. Temporal drift in platform‐trial control outcomes further underlines the need for robustness [19], and ESS has also been used to calibrate prior hyperparameters in Bayesian Phase I–II dose‐finding [20].

## 4 Critical Review of Section iv.A.1: Calibration to Type‐I Error Rate

Section IV.A.1 defines the approach of selecting posterior probability threshold c to control overall FWER, typically at 0.025 one‐sided, with c approximated by clinical trial simulation for complex designs [4]. This approach is directed at Bayesian methods used to facilitate complex adaptive designs rather than to synthesise external information. The rationale is well‐founded: control of the Type‐I error rate is a central principle for confirmatory trials set out in ICH E9 [21], and calibration enables Bayesian designs to satisfy it without philosophical overhaul. For noninformative‐prior adaptive designs—group sequential Bayesian frameworks, response‐adaptive randomisation, seamless designs—calibration is technically appropriate and practically important [22].

A conceptual tension remains. When calibrated to control frequentist FWER, the Bayesian decision rule is judged by a frequentist criterion: threshold c is determined by the design, the prior, and the null hypothesis used in the simulations, and is not a probability statement about efficacy. The posterior remains Bayesian in form, but the decision criterion is frequentist. Arjas and Gasbarra [23] argue that requiring a Bayesian design to meet a frequentist Type‐I error bound is a hybrid that discards key Bayesian benefits, and propose controlling false‐positive risk through posterior‐based measures instead. Ji [5] reviews this debate, noting that the practical impact of calibration is not yet clear, that Type‐I error is conventionally evaluated under a point null hypothesis whereas Bayesian analyses place a prior over effect sizes, and that critics consider that overlaying frequentist error‐rate constraints on Bayesian cutoffs can yield conservative decision thresholds. Direct posterior interpretation (Section IV.A.2) may therefore be better aligned with designs whose priors accurately reflect pre‐trial belief or external data, yet the Guidance does not delineate when each paradigm is preferred.

It is therefore recommended that the final guidance: (a) delineate FWER calibration (noninformative‐prior adaptive designs) from direct posterior interpretation (confirmatory designs with well‐justified informative priors); (b) recommend that the number of simulation replications be chosen to achieve a pre‐specified Monte Carlo precision for each reported operating characteristic, rather than applying a single fixed count across all settings. For a simulated rejection probability $\hat{p}$ based on N independent replications, the Monte Carlo standard error is $\mathrm{SE}\left(\hat{p}\right)=\sqrt{\hat{p}\left(1-\hat{p}\right)/N}$, and the number of replications needed for a required standard error ${\mathrm{SE}}_{\mathrm{req}}$ is $N=\hat{p}\left(1-\hat{p}\right)/{\mathrm{SE}}_{\mathrm{req}}^{2}$ (see [24], Table 6, Section 5.3, and Equation (1); [25] provide further general guidance on the design of simulation studies). For a one‐sided Type‐I error rate of 0.025, N=10,000 replications give $\mathrm{SE}\left(\hat{p}\right)\approx 0.0016$ (a 95% Monte Carlo interval of about ±0.003), so achieving $\mathrm{SE}\left(\hat{p}\right)<0.001$ requires N≳25,000. Because the standard error of an estimated probability is largest at 0.5, N=10,000 also bounds the Monte Carlo standard error of any simulated probability by 0.005 ([24], Section 5.3). The final guidance should recommend that sponsors report the target precision, the resulting replication count, and the achieved Monte Carlo standard error alongside every simulated operating characteristic. The Guidance itself refers only to a large number of simulated trials (Section IV.B.1) and specifies no replication count, and, to the author's knowledge, neither the Guidance nor the simulation‐methodology literature cited here prescribes a universal minimum. As a pragmatic orientation, N=10,000 is suggested as a lower bound for confirmatory Type‐I error assessment; this is the author's own recommendation, motivated by the precision arithmetic above, and is not a minimum recommended in the literature; (c) clarify whether FWER is evaluated under the pre‐specified prior or marginal to it; and (d) affirm that FWER calibration does not substitute for bias simulation when informative priors are employed.

## 5 Complementarity With Project Optimus and the Dose‐Optimisation Guidance

Project Optimus calls for characterisation of the full benefit–risk profile across dose levels for molecularly targeted agents, kinase inhibitors, antibody‐drug conjugates and immunotherapies—agents whose dose–response relationships are frequently non‐monotone and whose cumulative toxicities extend well beyond standard DLT windows [6, 7, 26, 27]. The August 2024 dosage‐optimisation guidance [27], which is nonbinding, reflects this emphasis, recommending early pharmacokinetic and pharmacodynamic data collection, a randomised parallel dose–response comparison of multiple dosages, attention to tolerability over longer treatment periods rather than short DLT windows alone, and model‐informed approaches to dose selection. Project Optimus acknowledges that the maximum tolerated dose paradigm developed for cytotoxic chemotherapy can yield unnecessarily high recommended dosages for modern targeted therapies [6, 7, 27]. This shift is complemented by recent work formalising an estimands framework for early‐phase dose‐optimisation trials in oncology, which clarifies the treatment effect of interest, defines the intercurrent events relevant to benefit–risk characterisation (e.g., toxicity‐driven dose modification, treatment switching and early discontinuation), and specifies the population‐level summary measure appropriate for optimal‐biological‐dose selection [28]; adopting such a framework alongside the Guidance's statistical recommendations would further sharpen the precision with which benefit–risk trade‐offs are estimated and reported.

The 2026 Bayesian Guidance supplies much of the statistical architecture needed to meet these recommendations. Dose–response modelling—Bayesian Emax models, for which FDA has issued a fit‐for‐purpose determination for empirically based implementations [29], and Bayesian logistic regression for dose–toxicity [30], alongside the frequentist MCP‐Mod framework [31, 32]—enables simultaneous inference across all doses. The simulation framework of Section IV.B is applicable to adaptive interim dose‐dropping rules. BHM shrinkage across dose levels can operationalise a totality‐of‐evidence approach. Phase‐crossing borrowing can support the seamless development approaches that the 2024 guidance describes. Despite this complementarity, Section III.F describes mainly toxicity‐only designs for MTD finding—model‐based (CRM [33], BLRM [30]) and model‐assisted (BOIN [34], mTPI [35] and mTPI2)—and while it also cites the efficacy–toxicity trade‐off design of Thall and Cook [36] and BOIN12 [37] as approaches to identifying optimised dosages, the draft text provides no operationalised evaluation criteria, simulation standards or specification of appropriate operating characteristics for them. This leaves room for further operational detail precisely where Project Optimus calls for the most innovation, since the toxicity‐only designs are structurally incapable of identifying the OBD.

## 6 Seamless Phase I/II and Efficacy–Toxicity Designs: Regulatory Overview

Identifying the OBD requires simultaneous modelling of toxicity and efficacy as functions of dose—a task for which single‐endpoint escalation designs are structurally inadequate [36, 38, 39]. The CRM [33, 40], extended to late‐onset toxicities [41], optimises over toxicity alone and cannot identify OBD. Thall and Cook's EffTox design [36] formalised the joint approach, specifying efficacy–toxicity trade‐off contours that define the desirability of each dose and selecting the most desirable dose among those satisfying prespecified acceptability criteria. BOIN12 [37] extends the BOIN framework [34]—whose local design under a non‐informative prior has received an FDA fit‐for‐purpose determination [42], a determination that does not extend to BOIN12—to incorporate efficacy through a utility, with a pre‐tabulated decision rule that is straightforward to specify in a protocol. Efficacy‐augmented designs including STEIN [43], BOIN‐ET [44], bivariate CRM variants [45], and the information‐theoretic design of Mozgunov and Jaki [46], which does not require monotonicity of the dose–efficacy relationship, further populate this class.

Seamless Phase I/II designs eliminate the protocol break between escalation and dose selection, enabling every enrolled patient's data to inform the Phase 2 dose decision—consistent with the emphasis of the dose‐optimisation guidance on integrating all available efficacy, safety and pharmacokinetic data in dose selection [6, 27]. Late‐onset toxicity and efficacy are accommodated by data‐augmentation methods for Phase I–II designs [47] and by TITE‐BOIN12 [48], and late‐onset toxicity alone by the time‐to‐event BOIN [49]; the odds‐ratio‐based Phase I/II design of Yin et al. [50] provides a further joint toxicity–efficacy approach. For combination therapies, the partial‐order CRM [51] and the BOIN combination design [52] offer additional flexibility. Bayesian adaptive platform trials such as I‐SPY 2 [53] illustrate the use of Bayesian adaptive methods in randomised Phase II settings.

A natural primary operating characteristic for these designs is OBD selection probability—the probability of recommending the dose with the highest true utility—evaluated across scenarios where the OBD lies at or well below the MTD, and scenarios where no dose achieves an acceptable profile [36, 39]. The Guidance's simulation framework (Section IV.B) is directly applicable; what could usefully be added is an explicit statement that FWER is not a meaningful criterion for dose‐finding designs and that OBD selection and overdose control probabilities are the appropriate evaluation endpoints. Table 2 presents the proposed regulatory taxonomy for Section III.F.

**TABLE 2:** Proposed regulatory taxonomy of Bayesian dose‐finding designs, their alignment with project optimus, and recommended regulatory actions for the final guidance.

Design class	Primary objective	Key Bayesian feature	Project optimus alignment	Recommended regulatoryaction
Toxicity‐only escalation (CRM, BLRM, BOIN, mTPI/mTPI2)	MTD identification	Sequential posterior on DLT–dose curve	Limited—single‐endpoint; not OBD‐capable	Retain current description; explicitly state FWER is not the appropriate operating criterion; clarify that OBD identification requires joint endpoint modelling
Efficacy–toxicity joint models (EffTox, BOIN12, BOIN‐ET)	OBD identification via utility maximisation	Joint bivariate posterior; utility surface over (toxicity, efficacy)	Strong—simultaneously models benefit–risk	Expand Section III.F description; specify OBD selection probability and overdose control probability as primary operating characteristics; require prospective simulation across diverse dose–response scenarios
Seamless Phase I/II (data‐augmentation EffTox, TITE‐BOIN12, STEIN)	Concurrent dose‐finding and activity confirmation	Phase‐boundary‐free Bayesian adaptive allocation	Very strong—OBD concurrent with early activity assessment	Address via dedicated supplementary guidance or an expanded stand‐alone section; specify audit‐trail, adaptive decision documentation and late‐onset endpoint handling requirements
Multi‐agent combination (POCRM, BOIN combination)	Optimal dose combination identification	2‐D dose–response surface; partial ordering	Moderate—addresses combination regimen dose optimisation	Reference existing combination dose‐finding literature; encourage pre‐submission meeting for novel combination designs
Adaptive platform designs (Bayesian platform designs with dose optimisation)	Multi‐indication dose optimisation with cross‐indication borrowing	BHM across indications; response‐adaptive randomisation	Very strong—dose optimisation at scale across indications	Coordinate with Master Protocol and platform trial guidance; specify BHM exchangeability validation and simulation requirements for borrowing across indications

Abbreviations: BHM, Bayesian hierarchical model; BLRM, Bayesian logistic regression model; BOIN, Bayesian optimal interval; CRM, continual reassessment method; DLT, dose‐limiting toxicity; ESS, effective sample size; FWER, familywise Type‐I error rate; MTD, maximum tolerated dose; mTPI, modified toxicity probability interval; mTPI2, modified toxicity probability interval 2; OBD, optimal biological dose; POCRM, partial‐order continual reassessment method; STEIN, simple toxicity and efficacy interval; TITE, time‐to‐event.

A fundamental regulatory question raised by this gap is whether guidance on seamless Phase I/II and efficacy–toxicity joint model designs should be incorporated within the current Bayesian methodology guidance document or addressed in a dedicated supplementary guidance. The FDA has precedent for both approaches: the current Guidance addresses a broad range of Bayesian applications, whereas dedicated documents such as the 2024 dosage‐optimisation guidance [27] provide operationally specific standards for targeted methodological challenges. Given the complexity of seamless designs—including late‐onset endpoint handling, real‐time adaptive decision documentation, and the absence of a meaningful FWER criterion—a dedicated supplementary guidance, developed in coordination with Project Optimus, would allow for the depth and operational specificity that these designs require without disrupting the coherence of the existing Bayesian framework document. Irrespective of the regulatory vehicle chosen, the taxonomy in Table 2 and the operating characteristic standards described here are applicable. Sponsors may raise this question with FDA in Type C or pre‐IND/BLA meetings, including, where a design qualifies as a complex innovative trial design (CID), through the pathways described in FDA's guidance on interacting with the Agency on such designs [54], which notes that additional Type C meetings may be granted when a proposal relies on extensive simulations to discuss specific protocol implementations.

## 7 Documentation, Computation and Reproducibility

The Guidance's documentation expectations (Sections VII and VIII)—pre‐specified plans, prior and likelihood disclosure, posterior summaries with credible intervals, sensitivity analyses, shared code and simulation reports, MCMC settings and convergence diagnostics, and, in the reporting of results, the software used including version details and the seed numbers used for chain initiation [4]—are well suited to regulatory reproducibility needs, and pre‐specification also limits analytic flexibility [55]. Two points remain. First, the draft already asks for software version details and MCMC seed numbers (Section VIII.B) and for simulation code (Section VIII.A), while stating that FDA does not require any specific software (Section VII). It does not, however, itemise the wider computing environment (e.g., operating system and package dependencies), nor does it extend the seed and version expectations explicitly to the simulation studies that generate operating characteristics. Because version‐specific behaviour changes in Stan, JAGS and SAS PROC MCMC create reproducibility risk, the final guidance could encourage documentation of the full computational environment for both the primary analysis and the simulation studies, for example through containerised environments (e.g., Docker). Second, for seamless Phase I/II designs, the final guidance should specify how real‐time interim analyses are documented in the SAP and how adaptive decision audit trails are maintained for regulatory review.

## 8 Overall Assessment and Priority Recommendations

The FDA's 2026 draft Guidance on the use of Bayesian methodology in clinical trials is a historic contribution to regulatory statistics. Its principal strengths are: a pluralistic, context‐sensitive framework for Bayesian success criteria; detailed treatment of prior specification and Bayesian borrowing illustrated with regulatory examples; explicit expectations for simulation and operating‐characteristic reporting; and comprehensive documentation standards. Its publication may also have international implications: it is consistent with the emphasis of EMA's regulatory science strategy on fostering innovative approaches to clinical trials [56], which may reduce uncertainty for global sponsors. Five priority recommendations for the final guidance are summarised in Table 3.

**TABLE 3:** Priority recommendations for the final FDA guidance on Bayesian methodology.

Section	Current limitation	Recommended enhancement
III.F	Detailed description of MTD‐focused designs; efficacy–toxicity designs (Thall–Cook, BOIN12) cited for optimised dosages without operationalised evaluation criteria, and seamless Phase I/II designs not discussed as such	Incorporate regulatory taxonomy (Table 2); specify OBD selection probability as primary operating characteristic; state FWER is not the criterion for dose‐finding designs
IV.A.1	Does not delineate when FWER calibration is preferred over direct posterior interpretation; refers to a large number of simulated trials without a precision criterion	Specify conditions for FWER calibration vs. direct posterior interpretation; recommend replication counts sized to a pre‐specified Monte Carlo precision target (e.g., N≈25,000 for MCSE <0.001 near α=0.025; N=10,000 yields MCSE ≈0.0016), with the achieved Monte Carlo error reported alongside each operating characteristic (N=10,000 is suggested by the author as a pragmatic lower bound); clarify prior‐marginal vs. conditional FWER
V.D–E	Dynamic discounting (commensurate and supervised power priors) named in Section V.D.4 without citation of the primary literature; guidance on selecting and validating the adaptivity mechanism is limited	Point to foundational dynamic‐borrowing references and recommend them for consideration when exchangeability is uncertain; recommend a pre‐specified adaptivity mechanism and simulation‐based bias and MSE assessment
V.E	ESS and prior mean offered as prior‐influence metrics; effect of a mislocated high‐ESS prior on bias and MSE not addressed	Clarify that ESS and prior mean describe the prior, not its consequences for estimation; recommend simulation‐based bias and MSE evaluation alongside them to detect mislocated priors
VII–VIII	Software versions and MCMC seeds requested (VIII.B); computing environment and simulation‐study seeds not itemised	Extend version and seed documentation to simulation studies and, where feasible, the computing environment (e.g., operating system, dependencies); encourage containerised computing; specify audit‐trail requirements for adaptive decision points

## 9 Conclusion

The FDA's 2026 draft guidance on the use of Bayesian methodology in clinical trials is a landmark regulatory document providing the clarity sponsors and statisticians have long sought for Bayesian primary analyses in drug development. Its treatment of prior specification, Bayesian borrowing, operating characteristics and documentation is comprehensive and will elevate the quality of Bayesian submissions across therapeutic areas.

However, the dose‐finding section offers limited operational detail for the OBD‐oriented designs that Project Optimus calls for. Efficacy–toxicity joint models—structurally capable of identifying OBD—are cited in Section III.F, and seamless Phase I/II designs are not discussed as such, with no operationalised evaluation criteria, simulation standards or specification of appropriate operating characteristics. Whether this gap is addressed by expanding Section III.F using the regulatory taxonomy proposed in Table 2 or by issuing a dedicated supplementary guidance document, the regulatory community requires clearer operational standards for these innovative designs. Expanding the description of efficacy–toxicity joint models and seamless Phase I/II designs, and clarifying conditions and simulation standards for Type‐I error calibration—including precision‐based replication recommendations [24, 25] and pointers to foundational dynamic‐borrowing methodology [15, 16]—would transform an already excellent document into a complete regulatory framework for 21st‐century Bayesian oncology trial design. The Guidance remains a draft at the time of writing, and the observations above refer to its January 2026 text. Sponsors and statisticians can engage with FDA on the issues raised here by scheduling Type C or pre‐IND/BLA meetings to discuss specific protocol implementations, through the pathways described in FDA's guidance on interacting with the Agency on complex innovative trial designs [54], or by contacting the relevant review division to propose alternative approaches. FDA's procedures also allow comments on guidance documents to be submitted at any time (21 CFR 10.115(g)(5)) under Docket FDA‐2025‐D‐3217.

## Funding

This research work was supported by Dr. Ayon Mukherjee's National Institute for Health and Care Research Grant (grant no: NIHR301614).

## Ethics Statement

The author has nothing to report.

## Consent

The author has nothing to report.

## Conflicts of Interest

Dr. Ayon Mukherjee is on the editorial board of the journal Pharmaceutical Statistics.

## Data Availability

Data sharing not applicable to this article as no datasets were generated or analysed during the current study.
